# Secure Cooperative Communications in 6G Networks: A Constrained Hierarchical Reinforcement Learning Framework with Hybrid Action Space

**DOI:** 10.3390/e28040412

**Published:** 2026-04-04

**Authors:** Xiaosi Tian, Zulin Wang, Yuanhan Ni

**Affiliations:** School of Electronic and Information Engineering, Beihang University, Beijing 100191, China; xiaosi_tian@buaa.edu.cn (X.T.); wzulin@buaa.edu.cn (Z.W.)

**Keywords:** hierarchical reinforcement learning, cooperative communication, secrecy capacity, relay selection, power allocation

## Abstract

With the rapid evolution toward 6G networks, ensuring robust physical layer security (PLS) in highly dynamic and heterogeneous wireless environments has become a key challenge. Traditional security methods often struggle to adapt to time-varying channels, especially in the absence of perfect channel state information. Furthermore, the dynamic nature of node selection and power allocation in heterogeneous networks creates a complex hybrid action space operating across multiple timescales, significantly complicating the design of efficient and adaptive security strategies. To address this, this paper proposes a novel constrained hierarchical reinforcement learning (CHRL) framework for secure cooperative communications in next-generation wireless systems. The framework is designed to optimize secrecy performance within a hybrid action space comprising both discrete node selection and continuous power allocation, operating at different timescales. By hierarchically decoupling the joint optimization problem, the upper layer performs risk-aware node selection to maximize long-term secrecy capacity (SC) while guaranteeing a stable and secure link. At the lower layer, we develop a constrained MiniMax Multi-objective Deep Deterministic Policy Gradient (M3DDPG) algorithm that optimizes power allocation considering worst-case conditions. Lagrange multipliers are integrated to enforce a strictly positive SC constraint throughout transmission, effectively preventing security outages. Simulation results under time-varying Rayleigh fading channels demonstrate that the proposed CHRL framework outperforms existing HRL methods, achieving up to 17% improvement in SC while strictly maintaining security constraints. These results validate the effectiveness of the proposed approach for enhancing PLS in next-generation cooperative wireless networks.

## 1. Introduction

Next-generation wireless systems envisioned for beyond-5G/6G networks are evolving toward highly dynamic and heterogeneous architectures characterized by ultra-dense device connectivity, diverse service requirements, and rapidly time-varying network topologies [1,2]. The coexistence of massive Internet-of-Things devices and high-mobility terminals significantly increases environment complexity. Consequently, this openness introduces severe challenges for secure confidential transmission [3]. Since conventional cryptographic mechanisms often struggle with real-time threats and channel uncertainty, physical layer security (PLS) has emerged as a vital solution. By exploiting the inherent randomness of wireless channels, PLS effectively enhances communication confidentiality in these complex scenarios [4,5].

Extensive studies have investigated PLS in communication systems [6,7,8]. One effective approach is to introduce relays and jammers as intermediate nodes in wireless communication networks [9,10,11]. Jammers transmit artificial interference to degrade the eavesdropping channel, while relays enhance the legitimate channel by forwarding source signals to the intended destination. In this way, the main channel can be strengthened relative to the eavesdropping channel, thereby improving the secrecy capacity (SC). To maximize secrecy performance, it is essential to select appropriate relays and jammers from multiple candidates and dynamically adjust their transmit power strategies. To achieve this, various optimization-based methods have been employed in the literature. For example, authors in [6] studied cooperative networks with selfish but friendly intermediate nodes and proposed a pricing-based relay and jammer selection algorithm to maximize SC. In [12], an optimal power allocation strategy was derived for simple multi-hop networks under system constraints. The work in [13] introduced an antenna selection scheme to leverage spatial diversity for enhanced security. Despite their contributions, these approaches critically depend on the availability of perfect channel state information (CSI).

However, the dynamic nature of wireless channels, often modeled as time-varying fading processes, introduces considerable uncertainty into the system [14,15,16]. Under such conditions, traditional optimization-based methods for node selection and power allocation become unreliable, as they generally need prior knowledge of the environment. Moreover, the joint optimization of node selection and power allocation creates a hybrid action space comprising both discrete and continuous variables that operates on different timescales. This hybrid action space at different timescales further complicates the design of efficient and adaptive security strategies [17].

Reinforcement learning (RL) offers a powerful model-free framework for addressing adaptive decision-making problems under uncertainty [18,19,20]. By continuously interacting with the environment, RL agents are able to learn transmission strategies without requiring explicit system models, making RL particularly attractive for dynamic and complex wireless networks [21]. In the application of RL in complex 6G heterogeneous scenarios [22], hierarchical RL (HRL) has been introduced to address the challenges of hybrid action space optimization, specifically involving joint node selection and power allocation for PLS [23,24]. By decomposing intricate resource allocation tasks into distinct sub-levels, this framework achieves superior convergence performance compared to conventional flat RL architectures [25,26,27]. In [25], a HRL approach is proposed, performing relay selection and power allocation at separate levels. However, a key limitation of these methods lies in the discretization of power allocation actions, which prevents the model from attaining theoretically optimal power values. Additionally, existing studies mainly focus on SC maximization [28] or secrecy outage probability minimization [25,28]. Most of these studies did not impose strict positive SC constraints, making it difficult to guarantee the requirement of stable secure communication.

Furthermore, conducting this research highlights several key challenges in designing adaptive security strategies to address these limitations. First, the joint optimization of topology management and resource allocation creates a complex hybrid action space. Directly applying conventional RL algorithms often leads to a combinatorial explosion, rendering the training process unstable and computation strictly infeasible for real-time deployment. Second, maintaining a strictly positive SC at all times is exceedingly difficult in highly dynamic environments where perfect instantaneous CSI is unavailable. Finally, achieving robust continuous power control requires striking a delicate balance in a competitive scenario. The cooperative jammers must sufficiently degrade the eavesdropping channels without inadvertently collapsing the legitimate transmission links, a multi-objective trade-off that is highly sensitive to parameter oscillations during model training.

To address the above challenges, this paper proposes a constrained HRL (CHRL) framework for hybrid action space optimization in secure cooperative communication. The main contributions of this paper are summarized as follows.

- Hybrid action hierarchical learning framework
  Different from conventional HRL approaches that discretize continuous variables or optimize a single decision layer, we develop a hybrid action hierarchical framework that jointly models discrete node selection and continuous power allocation across multiple timescales. The proposed framework not only resolves the combinatorial explosion problem inherent in hybrid discrete-continuous action spaces but also overcomes the performance degradation caused by power allocation discretization seen in conventional methods.
- Strict secrecy constraint enforcement
  Existing studies primarily focus on maximizing SC or minimizing outage probability, often failing to impose strict positive SC constraints. To address this, our framework enforces security guarantees across both decision levels. Our upper-level policy explicitly models secrecy violations as long-term risk signals, enabling a reward–risk coupled policy for discrete node selection that guarantees a strictly positive SC under dynamic channel variations. Complementing this, the lower-level policy employs a Lagrange multiplier-based constraint mechanism, ensuring that continuous power control is optimized while maintaining secure communication at all times.
- Constrained MiniMax continuous power allocation under worst-case channel uncertainty
  In jammer-assisted cooperative communication, the lower-level power allocation is inherently a competitive game: friendly jammers must degrade eavesdropping channels while minimizing their interference to legitimate receivers. Existing methods often fail to fully account for this delicate balance, leading to vulnerable strategies that severely compromise system robustness. To address this, we introduce a virtual adversary to represent worst-case channel conditions and the potential degradation of the legitimate link caused by jammers. By modeling this competitive scenario as a constrained zero-sum game, we deploy a constrained MiniMax Multi-objective Deep Deterministic Policy Gradient (M3DDPG) algorithm to achieve robust and strictly secure continuous power allocation at the physical layer.

In summary, the proposed framework jointly integrates hierarchical hybrid-action optimization, risk-aware node selection, and constrained minimax power control into a unified security-aware learning architecture. Numerical results show our framework outperforms existing methods, improving SC by 6%, 17%, and 49% over H-RL [25], hierarchical Q/E networks (H-Q/E) [23], and M3DDPG [29], respectively, while guaranteeing strict adherence to security constraints.

The rest of this paper is organized as follows. Section 2 presents the system model, including the Rayleigh time-varying channel and the cooperative communication setup with relays and jammers. Section 3 formulates the aforementioned secure cooperative communication scenario as a constrained Markov decision process (CMDP). Section 4 introduces the proposed CHRL framework, detailing the two-level structure for relay/jammer selection and power allocation. The experimental results are presented in Section 5 and the conclusion is drawn in Section 6.

## 2. System Model

In this section, we first establish a Rayleigh time-varying channel using stochastic differential equations (SDEs). Under this channel, we propose a secure cooperative wireless communication system with relays and jammers.

### 2.1. Rayleigh Time-Varying Channel Using SDEs

To better capture the characteristics of real-world wireless channels, paper [16] employs SDEs to model the random behavior of the signal envelope variations in the time domain. The in-phase and quadrature components, denoted as ${I(t),Q(t)}_{t\geq 0}$, are considered as Markov uncorrelated Gaussian random processes with time-varying mean and variance. Therefore, I(t) and Q(t) are solutions to the following identically distributed Ornstein–Uhlenbeck SDEs for t>0:

(1) $$\left\{\begin{array}{cc} dI\left(t\right)=-\frac{1}{2}BI\left(t\right)dt+\frac{\sqrt{2}}{2}B^{\frac{1}{2}}\sigma dW^{\left(I\right)}\left(t\right), & t>0,\\ dQ\left(t\right)=-\frac{1}{2}BQ\left(t\right)dt+\frac{\sqrt{2}}{2}B^{\frac{1}{2}}\sigma dW^{\left(Q\right)}\left(t\right), & t>0,\\ I\left(0\right)=I_{0}, & \\ Q\left(0\right)=Q_{0}, & \end{array}\right.$$

where B=2k and $\sigma =\frac{\beta }{\sqrt{k}}$. $W^{\left(I\right)}\left(t\right)$ and $W^{\left(Q\right)}\left(t\right)$ are independent Wiener processes. In this context, let g(t) be the Markovian projection of $g\left(t\right)=I^{2}\left(t\right)+Q^{2}\left(t\right)$, which is obtained by solving the following SDE [16]:

(2) $$\left\{\begin{array}{cc} dg(t) & =B\left(\sigma^{2}-g\left(t\right)\right)dt+\sigma \sqrt{2Bg(t)}dW\left(t\right),\\ g(0) & =g(0). \end{array}\right.$$

Using the Fokker–Planck equation, the limit distribution p(.) of the projected process g(t) satisfies:

(3) $$\begin{array}{c} \frac{\partial }{\partial y}\left(yp\left(y\right)\right)=\left(1-\frac{y}{\sigma^{2}}\right)p\left(y\right),\;y>0. \end{array}$$

The solution of Equation (3) is:

(4) $$p\left(y\right)=\frac{1}{\sigma^{2}}e^{\frac{-y}{\sigma^{2}}},\;y>0.$$

**Figure 1:**
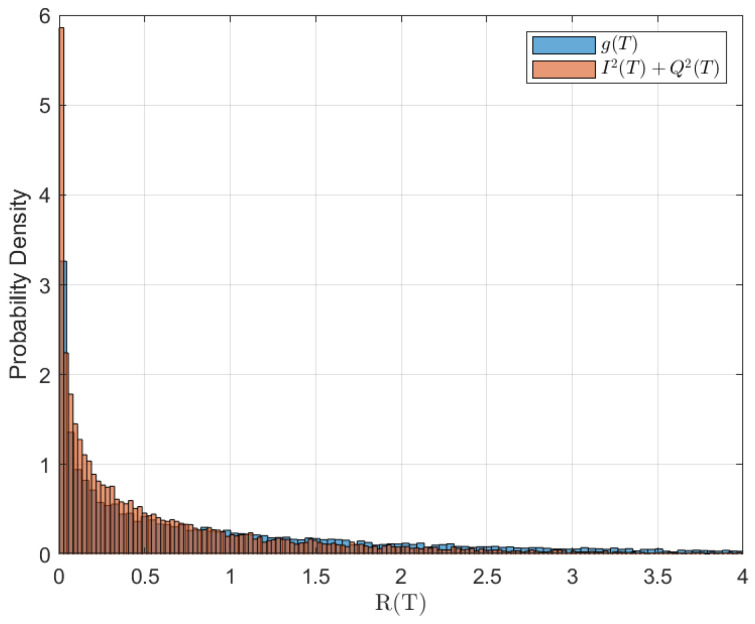
Rayleigh Fading: Histogram of g(T) and $I^{2}\left(T\right)+Q^{2}\left(T\right)$ using $10^{6}$ samples with the following parameters: T=4, N=100, $I_{0}=0$, $Q_{0}=0$, B=1, and σ=1.

Therefore, g(t) follows an asymptotic exponential distribution with parameter $\frac{1}{\sigma^{2}}$, which is the exact asymptotic distribution of g(t). We solved the original SDE Equation (1) and the projected SDE Equation (2), where *T* is the final time. The histograms of g(T) and $I^{2}\left(T\right)+Q^{2}\left(T\right)$ are compared in Figure 1. As shown in the figure, the samples of g(T) produce a histogram that closely resembles $I^{2}\left(T\right)+Q^{2}\left(T\right)$.

**Figure 2:**
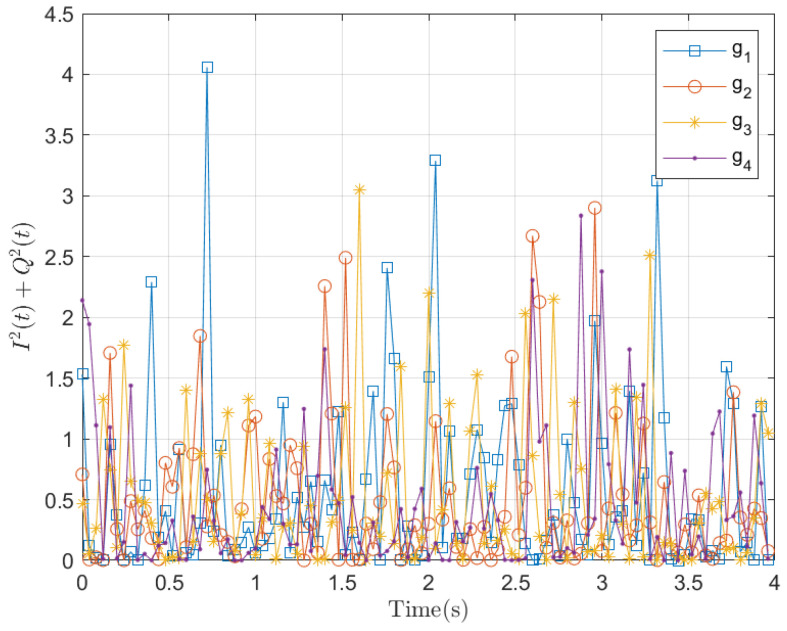
Samples of $I^{2}\left(t\right)+Q^{2}\left(t\right)$ with the following parameters: T=4, N=100, $I_{0}=0$, $Q_{0}=0$, B=1, and σ=1.

Additionally, Figure 2 shows the variation in time-varying Rayleigh channel coefficients modeled by the SDE over time. We note that the waveform manifests as randomly occurring pulses or spikes, with the amplitudes of these pulses adhering to an exponential distribution.

This channel modeling approach implies that the channel coefficients cannot be accurately predicted in advance during node selection and power allocation. Consequently, traditional optimization-based methods that rely on perfect CSI become inapplicable under this setting.

### 2.2. Multi-Hop Secure Cooperative Communication Framework

**Figure 3:**
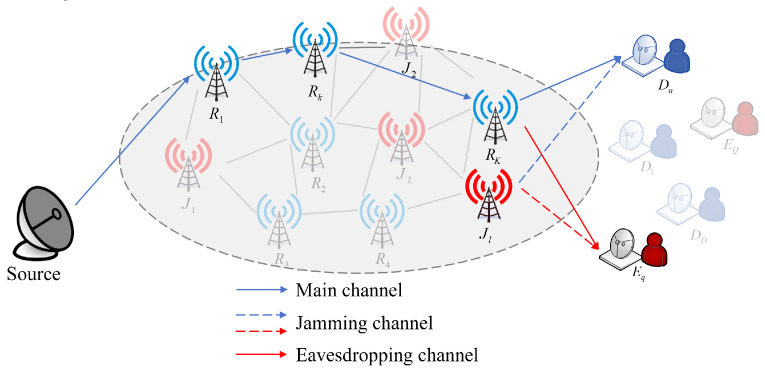
System model of cooperative communication.

We delineate a sophisticated multi-hop cooperative communication network architecture designed for secure information transmission in complex and dynamic environments, shown in Figure 3. The network resides in a wireless setting comprising a source node *S*, a set of *N* legitimate destination (Bobs) denoted by $D=\{D_{1},D_{2},\ldots ,D_{N}\}$, and a group of *Q* external eavesdroppers (Eves) represented by $E=\{E_{1},\ldots ,E_{Q}\}$. To facilitate reliable communication over extended distances and overcome channel fading, we consider a pool of *K* candidate nodes $R=\{R_{1},R_{2},\ldots ,R_{K}\}$, which serve as the relaying infrastructure. For each destination $D_{n}$, a specific multi-hop transmission path $P_{n}$ is established, represented by an ordered sequence of nodes $P_{n}=\left\{n_{0,n},n_{1,n},\ldots ,n_{M_{n},n}\right\}$, where $n_{0,n}=S$ is the source and $n_{M_{n},n}=D_{n}$ is the *n*-th destination. A defining characteristic of this model is the presence of simultaneous internal and external security threats, where the intermediate nodes ${\left\{n_{i,n}\right\}}_{i=1}^{M_{n}-1}\subset R$ are categorized as untrusted relays. These nodes are essential for forwarding confidential messages using a decode-and-forward (DF) protocol but also act as potential internal eavesdroppers. To counteract these threats, a subset of idle nodes J(t)⊂R is dynamically selected as cooperative jammers to transmit jamming signals to degrade the channels of both external and internal eavesdroppers. Regarding hardware and channel properties, all nodes are equipped with a single antenna and operate in half-duplex mode, while the wireless channels are modeled as independent Rayleigh time-varying fading processes.

To focus our analysis on the relaying links, we assume that there is no direct communication path from the source to Bobs and Eves. This means that the destination can only receive information from the source through the relay nodes, and the Eve cannot intercept any information from the broadcast channel between the source and the intermediate nodes [6]. Consequently, the SC is solely determined by the cooperative channel.

### 2.3. Multi-Hop Communication Process

The end-to-end information transmission from the source *S* to each designated destination $D_{n}$ is characterized by a sequential multi-hop transmission procedure structured into $M_{n}$ consecutive time slots. This process initiates with a broadcast phase, where the source node *S*, designated as $n_{0,n}$, transmits the confidential signal $x_{1}$ to the primary relay $n_{1,n}$ within the predefined path $P_{n}$. To mitigate the eavesdropping risk from both the *Q* Eves E and the idle untrusted relays within the pool R, a subset of nodes J is dynamically activated as cooperative jammers. These jammers emit jamming signal $s_{j}$ concurrently with the source’s broadcast, effectively degrading the signal-to-interference-plus-noise ratio (SINR) at any unauthorized tapping point while the first relay attempts to reliably decode the transmission.

Following the initial broadcast, the communication enters the intermediate relaying phases, which span from hop i=2 to $M_{n}-1$. Each time slot *i* within this sequence utilizes a DF protocol, where the *i*-th node in the path which acted as the receiver in the (i−1)-th slot first decodes the confidential message received previously. Upon successful decoding, this relay forwards the re-encoded signal to the subsequent node $n_{i,n}$ in the path sequence.

The final stage of the communication procedure is the delivery phase, occurring in the $M_{n}$-th time slot. In this phase, the final intermediate relay in the path $P_{n}$ transmits the processed signal to the ultimate destination $D_{n}$, designated as $n_{M_{n},n}$. Similar to the preceding hops, this concluding transmission is conducted under the protection of cooperative jamming to ensure that the cumulative SC is not compromised at the terminal link.

### 2.4. Signal Model at the *i*-th Hop

Within the established multi-hop network, the transmission from the source to each destination $D_{n}$ is decomposed into a sequence of discrete relaying intervals. We consider the *i*-th hop within the specific path $P_{n}$, where node $u=n_{i-1,n}$ serves as the active transmitter (either the source or a preceding relay) and $v=n_{i,n}$ serves as the intended legitimate receiver. In this complex wireless environment, the transmission is characterized by the dynamic interplay between the confidential signal, the artificial interference generated by cooperative jammers J, and ubiquitous environmental noise.

- Legitimate Signal Model
  During the *i*-th time slot, the signal received at the legitimate node *v* is modeled as a superposition of the desired information-bearing signal and the aggregated jamming signals designed to confuse potential eavesdroppers. The received signal $y_{v,i}^{\left(n\right)}$ is mathematically expressed as:
  (5) $$y_{v,i}^{\left(n\right)}=\sqrt{P_{u}}h_{u,v}x_{i}+\sum_{j\in J}\sqrt{P_{j}}h_{j,v}s_{j}+v_{v},$$
  where $P_{u}$ and $P_{j}$ denote the instantaneous transmit powers of the transmitting node and the *j*-th jammer, respectively. The term $h_{u,v}$ represents the complex channel fading coefficient between the transmitter and the receiver, while $h_{j,v}$ represents the channel coefficient between the *j*-th jammer and the receiver. $x_{i}$ is the normalized confidential message symbol. The summation term accounts for the deliberate interference $s_{j}$ emitted by the cooperative jammers, which serves to degrade the eavesdropping channels. Finally, $v_{v}∼\mathrm{CN}\left(0,\sigma^{2}\right)$ represents the additive white Gaussian noise at the receiver.
- External Eavesdropping Model
  To account for external security threats, we assume the presence of *Q* non-colluding Eves that monitor the transmission medium from various geographical locations. For any specific Eve $E_{q}$, the intercepted signal is subject to the same broadcast environment but experiences different channel fading:
  (6) $$y_{E_{q},i}^{\left(n\right)}=\sqrt{P_{u}}h_{u,E_{q}}x_{i}+\sum_{j\in J}\sqrt{P_{j}}h_{j,E_{q}}s_{j}+v_{E_{q}}.$$
  The effectiveness of the secrecy strategy depends on the ability of the jammers to ensure that the interference power at $E_{q}$ significantly outweighs the signal power, thereby minimizing the information leakage.
- Internal Untrusted Relay Eavesdropping
  A unique challenge in this network is the untrusted nature of the relay pool R. Any relay node $R_{k}\in R$ that is not explicitly assigned as the transmitter or receiver for the current hop *i* is regarded as a potential internal eavesdropper. These nodes may attempt to decode the message they are tasked to forward in other slots. The signal intercepted by an untrusted relay $R_{k}$ is modeled as:
  (7) $$y_{R_{k},i}^{\left(n\right)}=\sqrt{P_{u}}h_{u,R_{k}}x_{i}+\sum_{j\in J}\sqrt{P_{j}}h_{j,R_{k}}s_{j}+v_{R_{k}}.$$
  This modeling approach necessitates a sophisticated power allocation strategy. While increasing $P_{j}$ can effectively jam Eves, it may inadvertently interfere with the legitimate receiver *v* or provide varying levels of protection against different untrusted relays depending on their spatial distribution.

### 2.5. Secrecy Capacity Formulation

Based on the multi-destination and multi-eavesdropper signal models established in the previous section, the secrecy performance of each hop must be rigorously quantified to account for the heterogeneous risks posed by internal and external adversaries.

- Instantaneous Hop Channel Capacity
  For the *i*-th hop of the *n*-th path, where node *u* transmits to node *v*, the legitimate channel capacity $C_{v,i}^{\left(n\right)}$ is derived from the Shannon capacity formula, considering the interference from the cooperative jammers J:
  (8) $$C_{v,i}^{\left(n\right)}=\log_{2}\left(1+\frac{P_{u}g_{u,v}}{\sigma^{2}+\sum_{j\in J}P_{j}g_{j,v}}\right),$$
  where $g_{a,b}={\left|h_{a,b}\right|}^{2}$ represents the instantaneous channel power gain between any two nodes *a* and *b* at time *t* modeled in Section 2.1.
  Simultaneously, the information leakage risk is characterized by the maximum achievable rate among all potential Eves. In this scenario, the total information leakage rate $C_{leak,i}^{\left(n\right)}$ is determined by the most favorable channel conditions available to either Eves or idle untrusted relays:
  (9) $$C_{leak,i}^{\left(n\right)}=\max\left\{\max_{q\in E}\log_{2}\left(1+\Gamma_{E_{q},i}^{\left(n\right)}\right),\max_{R_{k}\in R_{\left\{u,v\right\}}}\log_{2}\left(1+\Gamma_{R_{k},i}^{\left(n\right)}\right)\right\},$$
  where $\Gamma_{E,i}^{\left(n\right)}$ and $\Gamma_{int,i}^{\left(n\right)}$ denote the corresponding SINR at the Eve $E_{q}$ and the idle untrusted relay $R_{k}$, respectively.
- End-to-End Secrecy Capacity
  Due to the utilization of the DF protocol across the multi-hop architecture, the end-to-end performance is subject to the inter-hop performance interdependencies. Specifically, the end-to-end transmission rate is restricted by the link with the minimum instantaneous capacity, often referred to as the restrictive throughput constraint of the multi-hop chain. Consequently, the end-to-end SC for destination $D_{n}$, denoted as $C_{S,n}$, is defined as the difference between the bottleneck legitimate rate and the peak leakage rate encountered along the entire path:
  (10) $$C_{S,n}=\min_{i\in \{1,\ldots ,M_{n}\}}C_{v,i}^{\left(n\right)}-\max_{i\in \{1,\ldots ,M_{n}\}}C_{leak,i}^{\left(n\right)},$$
- SC Maximization Problem
  The ultimate goal of the system is to determine an optimal policy π that maximizes the expected discounted sum of SC over the entire communication duration *T* for all *N* Bobs while strictly adhering to security and power constraints:
  (11a) $$\begin{array}{cc} \max_{\pi }\; & E_{\pi }\left[\sum_{t=0}^{T}\gamma^{t}\sum_{n=1}^{N}C_{S,n}\left(t\right)\right] \end{array}$$
  (11b) $$\begin{array}{cc} s.t.\; & C_{S,n}\left(t\right)>0,\forall n,\forall t, \end{array}$$
  (11c) $$\begin{array}{cc} \; & 0\leq P_{u}\left(t\right)\leq P_{max,u},\;0\leq P_{j}\left(t\right)\leq P_{max,j},\forall t, \end{array}$$
  where γ∈[0,1] is the discount factor representing the importance of future rewards. This formulation ensures that the proposed framework not only maximizes throughput but also maintains a persistent security guarantee across the entire multi-hop network.

## 3. Constrained Markov Decision Process Formulation

In this section, the secure resource allocation problem in multi-hop, multi-destination networks is modeled as a CMDP. This framework is essential because traditional MDPs cannot inherently guarantee the strict requirements $C_{S,n}\left(t\right)>0$ necessary for PLS. The CMDP is defined by the quintuple (S,A,P,π,r,c), where S is the state space, A is hybrid action space, P is the state transition probability, π is the policy, *r* is the reward function, and *c* is the cost function.

### 3.1. Comprehensive State Space (S)

The state space $s_{t}\in S$ is designed to provide a Markovian representation of the environment, encapsulating the high-dimensional CSI and the hardware-specific constraints of the pool. The state vector at time *t* is defined as:

(12) $$s_{t}=\left\{G_{leg}\left(t\right),G_{int}\left(t\right),G_{ext}\left(t\right),P_{max},\Phi \left(t\right)\right\}.$$

The specific expressions for each component of Equation (12) are detailed as follows:

- Legitimate Channel Gain Set $G_{leg}\left(t\right)$
  This set contains the instantaneous channel power gains for every hop in the predefined paths $P_{n}$ for all *N* Bobs. In the RL setting, the agent cannot access instantaneous CSI and must rely on delayed CSI from the previous time slot. Thus, the state space is defined as the historical channel gains between all node pairs, expressed as:
  (13) $$G_{leg}\left(t\right)=\left\{g_{n_{i-1,n},n_{i,n}}\left(t-1\right)|n=1,\ldots ,N;i=1,\ldots ,M_{n}\right\},$$
  where $g_{u,v}\left(t\right)={\left|h_{u,v}\left(t\right)\right|}^{2}$ is the gain between node *u* and *v* modeled in Section 2.1. The total dimension of this set is $\sum_{n=1}^{N}M_{n}$, representing the full connectivity status of the legitimate network.
- Internal Leakage Channel Matrix $G_{int}\left(t\right)$
  This component characterizes the channel gains between the current transmitters and all potential internal eavesdroppers. For a transmitter *u* in the *i*-th hop, it is defined as:
  (14) $$G_{int}\left(t\right)=\left[g_{u,R_{k}}\left(t-1\right)\right]\in R^{1\times |R_{\left\{u,v\right\}}|},$$
  where $R_{k}\in R$ and $R_{k}\neq u,v$. This allows for the agent to evaluate the risk of information interception by the very nodes meant to assist the network.
- External Leakage Channel Matrix $G_{ext}\left(t\right)$
  This matrix captures the channel gains from both the active transmitters and the selected jammers to the *Q* Eves:
  (15) $$G_{ext}\left(t\right)=\left[\begin{array}{cccc} g_{u,E_{1}}\left(t-1\right) & g_{u,E_{2}}\left(t-1\right) & \ldots & g_{u,E_{Q}}\left(t-1\right)\\ g_{j_{1},E_{1}}\left(t-1\right) & g_{j_{1},E_{2}}\left(t-1\right) & \ldots & g_{j_{1},E_{Q}}\left(t-1\right)\\ \vdots & \vdots & \ddots & \vdots \\ g_{j_{\left|J\right|},E_{1}}\left(t-1\right) & g_{j_{\left|J\right|},E_{2}}\left(t-1\right) & \ldots & g_{j_{\left|J\right|},E_{Q}}\left(t-1\right). \end{array}\right]$$
  This (|J|+1)×Q matrix is vital for the lower-level controller to balance jamming power against signal leakage.
  It should be noted that neither Eves nor Bobs can access exact instantaneous CSI during decision-making due to the highly dynamic time-varying Rayleigh fading. Instead, the agent can only observe the delayed CSI from the previous time slot [30,31], which is treated as part of the environment state. This modeling choice maintains the Markov property while avoiding the unrealistic assumption of obtaining instantaneous CSI from Eves.
- Heterogeneous Power Constraint Vector $P_{max}$
  This is a static hardware-profile vector that informs the agent of the physical limitations of each node in the pool:
  (16) $$P_{max}={\left[P_{max,R_{1}},P_{max,R_{2}},\ldots ,P_{max,R_{K}}\right]}^{T}.$$
  Including this in the state space allows for the RL model to generalize its power allocation policy across nodes with different battery levels or hardware capabilities.
- Topology and Progress Indicator Φ(t)
  This vector tracks the real-time status of the multi-hop process and node availability:
  (17) $$\Phi \left(t\right)=[i_{1}\left(t\right),i_{2}\left(t\right),\ldots ,i_{N}\left(t\right),I_{occ}\left(t\right)],$$
  where $i_{n}\left(t\right)\in \left\{1,\ldots ,M_{n}\right\}$ is the current hop index for destination $D_{n}$. $I_{occ}\left(t\right)\in {\left\{0,1\right\}}^{K}$ is the node occupancy vector, where $I_{k}=1$ indicates that relay $R_{k}$ is currently busy forwarding data and cannot be selected as a jammer.

### 3.2. Hierarchical Action Space A

Due to the combinatorial complexity of selecting optimal paths and jammers alongside the continuous nature of power control, the action space is partitioned into two distinct sub-spaces.

- Discrete Joint Relay and Jammer Selection ($a_{t}^{D}$)
  The upper-level policy, acting as a meta-controller, manages the network topology by making discrete selections from the candidate pool R. The action is a composite decision process:
  Relay Selection: Based on the delayed legitimate channel gain set $G_{leg}\left(t\right)$, the agent identifies the optimal relay nodes $\left\{n_{i,n}\right\}$ to form the multi-hop transmission path $P_{n}$ for each Bob.
  Jammer Selection: From the subset of idle nodes F(t)⊂R, where $I_{k}=0$ in the node occupancy vector, the policy selects a binary vector $z_{t}\in {\left\{0,1\right\}}^{K}$ to designate cooperative jammers J(t). These nodes are strategically chosen to degrade the SINR at both external and internal eavesdropping points.
- Continuous Power Allocation ($a_{t}^{C}$)
  The lower-level policy determines the precise power levels for the selected relays and jammers. The action is a continuous vector $p_{t}=\left[P_{u}\left(t\right),P_{j,1}\left(t\right),\ldots ,P_{j,|J|}\left(t\right)\right]$, where $P_{u}$ is the power of the active transmitter in path $P_{n}$ and $P_{j}$ represents the power of the *j*-th jammer.

### 3.3. Transition Probability (P)

The transition probability $P(s_{t+1}|s_{t},a_{t})$ defines the evolution of the environment. In the context of a multi-hop secure network, this transition is governed by two distinct processes.

- Wireless Channel Dynamics
  The channel gains $G_{leg}\left(t\right),G_{int}\left(t\right),$ and $G_{ext}\left(t\right)$ are governed by independent stochastic processes. While these transitions follow the temporal correlation defined by SDEs, they remain exogenous and highly dynamic, rendering the instantaneous channel power gains unpredictable for the agent at the time of decision-making. Consequently, the agent must rely on delayed CSI from the previous time slot.
- Protocol-Driven State Evolution
  The topology and progress indicator Φ(t) evolves deterministically based on the agent’s actions. When a transmission for destination $D_{n}$ successfully completes a hop, meaning that the SC constraint is satisfied and the message is correctly decoded, the hop index $i_{n}$ increments from *i* to i+1. Once $i_{n}=M_{n}$, the node occupancy vector $I_{occ}$ is updated to release the nodes associated with that path back into the pool R.

### 3.4. Policy Mapping (π)

Due to the hybrid nature of the action space (discrete selection and continuous power), the policy π is decomposed into a hierarchical structure $\pi =\{\pi^{upper},\pi^{low}\}$.

- Upper-Level Policy: Node Selection ($\pi^{upper}$)
  The upper-level policy, typically governed by a discrete RL agent, maps the current state $s_{t}$ to the discrete jammer selection action $a_{t}^{D}$:
  (18) $$\pi^{upper}\left(s_{t}\right)\to z_{t},\;z_{t}\in {\left\{0,1\right\}}^{K}.$$
  This policy focuses on the long-term topological security of the network. It identifies which idle untrusted relays $R_{k}$ are strategically positioned to provide the most effective jamming for the current set of active hops while ensuring they are not wasted on hops that already have high intrinsic security.
- Lower-Level Policy: Power Allocation ($\pi^{low}$)
  Given the state $s_{t}$ and the discrete action $z_{t}$ chosen by the upper level, the lower-level policy determines the continuous power levels:
  (19) $$\pi^{low}\left(s_{t},z_{t}\right)\to p_{t},\;p_{t}=\left[P_{u}\left(t\right),P_{j,1}\left(t\right),\ldots ,P_{j,|J|}\left(t\right)\right].$$

### 3.5. Reward and Cost Functions (r,c)

The objective of the agent is not merely to maximize SC, but to do so within the safe operating region of the network.

- Objective Reward ($r_{t}$)
  The primary reward is the sum of the end-to-end SC across all Bobs. It incentivizes the agent to maximize the gap between the bottleneck legitimate rate and the peak leakage rate:
  (20) $$r_{t}=\sum_{n=1}^{N}C_{S,n}\left(t\right).$$
- Security Cost ($c_{t}$)
  To enforce the constraint $C_{S,n}\left(t\right)>0$ and to satisfy the physical transmission constraints of nodes, we define a set of cost functions consisting of three independent dimensions. This multi-dimensional cost mechanism is designed to apply precise quantitative penalties to any state–action pairs that induce information eavesdropping or exceed the power limits of transmitters and jammers, thereby enforcing the security and physical stability of the system within the algorithmic framework.
  Specifically, this multi-dimensional cost function set includes the following three core dimensions:
  Security Outage Cost ($c_{1,t}$): Specifically used to monitor and penalize decisions that lead to information eavesdropping. For any destination node $D_{n}$, this cost is defined using the mathematical indicator function I(·) as:
  (21) $$c_{1,t}^{\left(n\right)}=I\left(C_{S,n}\left(t\right)\leq 0\right).$$
  Transmitter Power Limit Cost ($c_{2,t}$): Used to constrain the transmission power of the active transmitter node *u* so that it does not exceed its hardware physical limit $P_{\max,u}$. It is defined as the truncation amount exceeding the maximum power threshold:
  (22) $$c_{2,t}=\max\left(0,P_{u}-P_{\max,u}\right).$$
  Jammer Power Limit Cost ($c_{3,t}$): Similarly, used to constrain the transmission power of the cooperative jammer *j* so that it does not exceed its hardware upper limit $P_{\max,j}$. It is defined as:
  (23) $$c_{3,t}=\max\left(0,P_{j}-P_{\max,j}\right).$$

## 4. Constrained Hierarchical Reinforcement Learning Framework

Building upon the CMDP established in the previous section, this section proposes a novel CHRL framework to address the multi-objective optimization problem in the proposed secure cooperative networks.

### 4.1. Framework Overview

**Figure 4:**
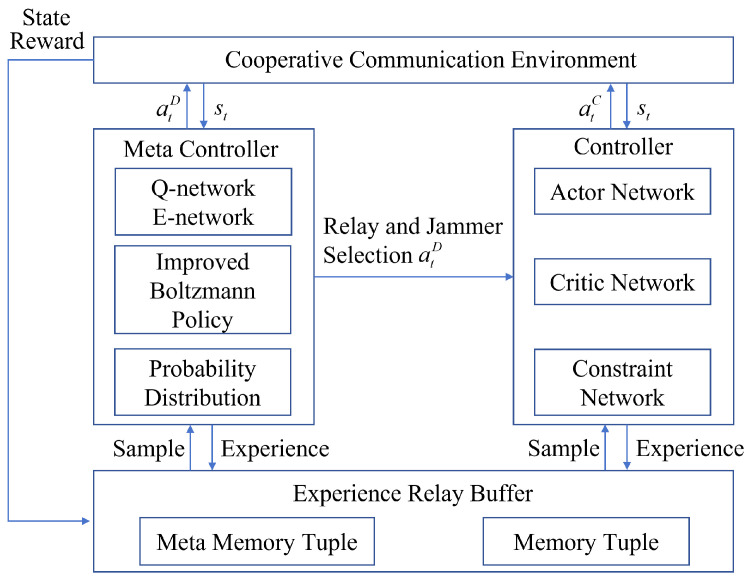
The proposed constrained hierarchical reinforcement learning (CHRL) framework for node selection and power allocation.

To address the joint optimization of node selection and power control in a multi-hop, multi-destination network, the CHRL framework is proposed, which is illustrated in Figure 4. This framework decomposes the original mixed-integer non-convex optimization problem into a two-level hierarchy on two distinct timescales. The upper-level manager, acting as a meta-controller, makes macro-decisions regarding node selection every *n* time steps. The lower-level executor, functioning as a controller, operates at each time step to make micro-decisions on precise power allocation based on the upper level’s macro-instruction and the current channel state. This decomposition effectively reduces the combinatorial complexity of the action space and enables more efficient exploration.

### 4.2. Upper Level: Risk-Aware Node Selection Policy

The upper-level controller functions as a strategic coordinator, responsible for selecting the optimal subset of cooperative jammers J(t) from the pool of idle untrusted relays R.

The upper-level policy $\pi^{upper}$ focuses on the macro-management of the network topology. It selects the jammer set J to maximize the expected long-term return $V^{upper}\left(s\right)$. The policy is defined as:

(24) $$y_{t}^{Q^{upper}}=\sum_{i=0}^{n}\gamma^{i}r_{t}+\gamma^{n}\max_{a_{t+n}^{D^{\prime }}}Q^{upper}\left(s_{t+n},a_{t+n}^{D^{\prime }};\theta^{Q^{upper^{\prime }}}\right),$$

where the discrete action $a_{t}^{D}$ represents the selection of relays and jammers. A Q-value function $Q^{upper}\left(s_{t},a_{t}^{D}\right)$ estimates the long-term expected SC when taking a particular node selection action in a given state. It is updated using a temporal difference (TD) error based on the aggregate reward $r_{t}$. The target value $y_{t}^{Q}$, the loss function $L(\theta^{Q})$, and the update of network parameter $\theta_{Q}^{upper}$ are defined as:

(25) $$\begin{array}{cc} y_{t}^{Q^{upper}} & =\sum_{i=0}^{n}\gamma^{i}r_{t+i}+\gamma^{n}\max_{a_{t+n}^{D^{\prime }}}Q^{upper}\left(s_{t+n},a_{t+n}^{D^{\prime }};\theta^{Q^{{upper}^{\prime }}}\right), \end{array}$$

(26) $$\begin{array}{cc} L(\theta^{Q^{upper}}) & =E\left[{\left(y_{t}^{Q^{upper}}-Q^{upper}\left(s_{t},a_{t}^{D};\theta^{Q^{upper}}\right)\right)}^{2}\right], \end{array}$$

(27) $$\begin{array}{cc} \theta^{Q^{upper}} & \leftarrow \theta^{Q^{upper}}-\alpha \nabla_{\theta^{Q^{upper}}}L\left(\theta^{Q^{upper}}\right). \end{array}$$

$\gamma^{n}$ is the discounted factor over *n* time steps, reflecting the macro-timescale of the meta-controller. $\theta^{Q^{upper^{\prime }}}$ is the target Q-network used to stabilize training. α is the network learning rate.

Complementarily, an E-value function $E^{upper}\left(s_{t},a_{t}^{D}\right)$ estimates the long-term risk exposure, specifically quantifying the probability of violating the security constraint ($C_{S,n}\left(t\right)\leq 0$) over time. The E-values are updated based on the *n* future risk values as:

(28) $$\begin{array}{cc} y_{t}^{E^{upper}} & =\sum_{i=1}^{n}\gamma^{i}c_{t+i}+\gamma^{n}\max_{a_{t+n}^{D^{\prime }}}E^{upper}\left(s_{t+n},a_{t+n}^{D^{\prime }};\theta^{E^{upper^{\prime }}}\right), \end{array}$$

(29) $$\begin{array}{cc} L(\theta^{E}) & =E\left[{\left(y_{t}^{E}-E^{upper}\left(s_{t},a_{t}^{D};\theta^{E^{upper}}\right)\right)}^{2}\right], \end{array}$$

(30) $$\begin{array}{cc} \theta^{E^{upper}} & \leftarrow \theta^{E^{upper}}-\alpha \nabla_{\theta^{E^{upper}}}L\left(\theta^{E^{upper}}\right). \end{array}$$

An improved Boltzmann policy integrates both estimates to prioritize actions with high reward and low risk. To select the discrete action $a_{t}^{D}$, the outputs of both networks are fused into a risk-aware probability distribution. The probability $P(a_{i})$ for a specific jammer combination $a_{i}$ is calculated as:

(31) $$P\left(a_{i}^{D}\right)=\frac{\exp\left(\frac{Q^{upper}\left(s_{t},a_{i}^{D}\right)-\sum \lambda_{n}E^{upper}\left(s_{t},a_{i}^{D}\right)}{\tau }\right)}{\sum_{a_{j}^{D}\in a_{t}^{D}}\exp\left(\frac{Q^{upper}\left(s_{t},a_{j}^{D}\right)-\sum \lambda_{n}E^{upper}\left(s_{t},a_{j}^{D}\right)}{\tau }\right)},$$

where τ>0 serves as a temperature parameter that regulates the exploration–exploitation trade-off.

### 4.3. Lower Level: Constrained Power Allocation Policy

The lower-level power allocation problem is inherently a competitive scenario, where the friendly jammer’s signal acts as a double-edged sword: it degrades the Eve’s channel while simultaneously causing potential interference to the legitimate receiver. To address this challenge, we model the power allocation as a constrained zero-sum game between the communication system and a virtual adversary that represents the worst-case channel conditions. This formulation aligns with the M3DDPG framework, whose core objective is to maximize the expected return under the most pessimistic assumptions about the environment or the opponent’s behavior.

The original constrained optimization problem Equation (11a) is reformulated using Lagrange multipliers λ to penalize constraint violations, leading to the Lagrangian dual problem:

(32) $$\min_{\lambda \geq 0}\max_{\pi }L\left(\pi ,\lambda \right),$$

where $\lambda ={\left[\lambda_{1},\lambda_{2},\lambda_{3}\right]}^{T}$, and the Lagrangian L(π,λ) is given by:

(33) $$L\left(\pi ,\lambda \right)=E_{\pi }\left[\sum_{t=0}^{T}\gamma^{t}\left(\underset{\mathrm{Reward}}{\underset{︸}{r_{t}}}-\sum_{i=1}^{C}\lambda_{i}\;\underset{{\mathrm{Cost}}_{i}}{\underset{︸}{c_{i,t}}}\right)\right].$$

The number of constraints is set to C=3 in this paper, following Equations (21)–(23). This formulation aligns with the M3DDPG’s MiniMax objective Equation (11a) by interpreting the penalized costs as adversarial contributions to the reward function, effectively training the agent against worst-case constraint violations.

The constrained M3DDPG architecture employs an Actor network $\mu (s_{t}|\theta^{\mu })$ to generate power allocation actions [29] and a Critic network $Q(s_{t},a_{t}^{C}|\theta^{Q})$ that estimates state–action values using TD learning. To align with the MiniMax objective, the target value $y_{t}$ is calculated by minimizing over a virtual adversary’s perturbation ψ to simulate worst-case conditions:

(34) $$y_{t}=\left(r_{t}-\sum_{i=1}^{C}\lambda_{i}c_{i,t}\right)+\gamma \min_{\psi }Q^{\prime }\left(s_{t+1},\mu^{\prime }\left(s_{t+1}|\theta^{\mu^{\prime }}\right)+\psi |\theta^{Q^{\prime }}\right).$$

The network parameters $\theta^{Q}$ are updated by minimizing the loss:

(35) $$\begin{array}{cc} L(\theta^{Q}) & =E\left[{\left(y_{t}-Q\left(s_{t},a_{t}^{C}|\theta^{Q}\right)\right)}^{2}\right], \end{array}$$

(36) $$\begin{array}{cc} \theta^{Q} & \leftarrow \theta^{Q}-\alpha \nabla_{\theta^{Q}}L\left(\theta^{Q}\right). \end{array}$$

Three separate Constraint networks $C_{i}\left(s_{t},a_{t}^{C}|\theta^{C_{i}}\right)$ estimate the expected cumulative future cost for each constraint i∈{1,2,3}. The target value $y_{c,i}$ is:

(37) $$y_{c,i}=c_{i,t}+\gamma C_{i}^{\prime }\left(s_{t},\mu^{\prime }\left(s_{t}|\theta^{\mu^{\prime }}\right)|\theta^{C_{i}^{\prime }}\right).$$

The parameters $\theta^{C_{i}}$ are updated by minimizing the mean squared error:

(38) $$\begin{array}{cc} L(\theta^{C_{i}}) & =E\left[{\left(y_{c,i}-C_{i}\left(s_{t},a_{t}^{C}|\theta^{C_{i}}\right)\right)}^{2}\right], \end{array}$$

(39) $$\begin{array}{cc} \theta^{C_{i}} & \leftarrow \theta^{C_{i}}-\alpha \nabla_{\theta^{C_{i}}}L\left(\theta^{C_{i}}\right). \end{array}$$

The Actor network $\mu (s_{t}|\theta^{\mu })$ determines the continuous power allocation vector $a_{t}^{C}$. The update rule performs gradient ascent on the Lagrangian objective, balancing the maximization of *Q* and the minimization of constraint costs $C_{i}$:

(40) $$\nabla_{\theta^{\mu }}J\approx \frac{1}{B}\sum_{j=1}^{B}\nabla_{\theta^{\mu }}\mu \left(s_{j}\right)\cdot \left[\nabla_{a}Q\left(s_{j},a\right)-\sum_{i=1}^{C}\lambda_{i}\nabla_{a}C_{i}\left(s_{j},a\right)\right]{|}_{a=\mu (S_{j})},$$

(41) $$\theta^{\mu }\leftarrow \theta^{\mu }+\alpha \nabla_{\theta^{\mu }}J,$$

where *B* is the batch size. In M3DDPG, the network is not updated using just the single most recent experience. Instead, a random set of transitions is sampled from the replay buffer $D_{low}$ to break the temporal correlation between consecutive steps. $s_{j}$ is the state component of the *j*-th transition in this random batch.

The multipliers are updated to enforce the constraints. If the expected violation estimated by the Constraint network is positive, the penalty weight $\lambda_{i}$ is updated by:

(42) $$\lambda_{i}\leftarrow \Gamma_{\Lambda }\left[\lambda_{i}+\eta_{\lambda }\cdot E\left[C_{i}\left(s_{t},\mu \left(s_{t}\right)\right)\right]\right],$$

where $\Gamma_{\Lambda }\left[x\right]=\max\left(0,x\right)$ ensures non-negativity.

### 4.4. Joint Learning Algorithm

The complete hierarchical learning algorithm integrates both levels through coordinated updates and experience sharing, as summarized in Algorithm 1.

**Algorithm 1** Joint CHRL for Secure Cooperative Communication**Require:** Environment, Network Architectures, Hyperparameters ($\gamma ,\tau ,\eta_{\lambda },B,\beta$)
  1:**Initialize:**  2:Upper-level networks: $Q^{upper},E^{upper}$ with weights $\theta^{Q^{upper}},\theta^{E^{upper}}$  3:Lower-level networks: Actor μ, Critic *Q*, Constraint Critics $C_{1},C_{2},C_{3}$ with weights $\theta^{\mu },\theta^{Q},\theta^{C_{i}}$  4:Target networks: $\theta^{\mu^{\prime }},\theta^{Q^{\prime }},\theta^{C_{i}^{\prime }}\leftarrow \theta^{\mu },\theta^{Q},\theta^{C_{i}}$  5:Replay buffers $D_{upper},D_{low}$  6:Lagrange multipliers $\lambda ={\left[\lambda_{1},\lambda_{2},\lambda_{3}\right]}^{T}\leftarrow 0$  7:**for** each iteration **do**  8: Initialize environment and observe initial state $S_{1}$  9: **for** t=1 to *T* **do**10:  **if** tmodn==1 **then**11:   Observe upper state $s_{t}$12:   Select discrete joint action $a_{t}^{D}$ by Equation (31)13:  **end if**14:  Construct lower state $s_{t}$15:  Select continuous power action $a_{t}^{C}$16:  Execute joint action $\left(a_{t}^{D},a_{t}^{C}\right)$17:  Observe reward $r_{t}$, constraint costs $c_{t}=\left[c_{1},c_{2},c_{3}\right]$, and next state $s_{t+1}$18:  Store transition $\left(s_{t},a_{t}^{C},r_{t},c_{t},s_{t+1}\right)$ in $D_{low}$19:  **if** samples sufficient in $D_{low}$ **then**20:   Sample random minibatch of *B* transitions from $D_{low}$21:   Update $\theta^{Q}$ by Equation (36)22:   Update $\theta^{C_{i}}$ by Equation (39)23:   Update $\theta^{\mu }$ by Equation (41)24:   Update Multipliers by Equation (42)25:   Soft Update Targets: $\theta^{\prime }\leftarrow \rho \theta +\left(1-\rho \right)\theta^{\prime }$ for all networks26:  **end if**27:  **if** tmodn==0 **then**28:   Store transition in $D_{upper}$29:   Update $Q^{upper}$ and $E^{upper}$ networks using stored experiences30:  **end if**31: **end for**32:**end for**

### 4.5. Computational Complexity Analysis

To demonstrate the practical feasibility of the proposed CHRL framework for real-time implementation in 6G cooperative networks, we quantitatively analyze its computational complexity from the perspectives of space dimensionality and time execution overhead [32] and compare its computational performance with traditional HRL methods [25].

Dimensionality Analysis: By employing hierarchical decoupling, the proposed framework successfully mitigates this combinatorial explosion. The upper-level discrete action space is reduced to O(K), and the lower-level continuous action space is reduced to O(1+|J|). Furthermore, the dimension of the comprehensive state space $s_{t}$, denoted as $D_{s}$, is defined by the concatenation of $G_{leg}\left(t\right)$, $G_{int}\left(t\right)$, $G_{ext}\left(t\right)$, $P_{max}$, and Φ(t). The total dimension evaluates to $D_{s}=\sum_{n=1}^{N}M_{n}+2K+N-2+\left(|J|+1\right)Q$.

Time Complexity Analysis: Assuming that all employed neural networks consist of *L* hidden layers with *H* neurons per layer, the computational time complexity must be evaluated separately for the offline training and online execution phases due to the nature of the Actor–Critic architecture.

During the offline training phase, the algorithm updates all seven networks (upper *Q*, upper *E*, lower Actor μ, lower Critic *Q*, and three Constraint networks $C_{i}$) via backpropagation using minibatches of size *B*. The computational overhead per training step is approximately $O(B\;\times \;[D_{s}H+LH^{2}+H(K+|J|)])$. This intensive computation is designated to be executed on centralized base stations or cloud servers a priori.

Conversely, during the online execution phase, the system only requires forward propagation to make real-time decisions. Crucially, the lower Critic and Constraint networks are strictly bypassed, as they solely serve as evaluators during training. The active networks during inference are limited to the upper macro-controllers and the lower Actor network. Consequently, the online execution complexity is drastically condensed to $O(D_{s}H+LH^{2}+H(K+|J|))$. This polynomial-time complexity firmly establishes that the proposed CHRL framework imposes minimal computational delay during online deployment, guaranteeing rapid adaptation to the highly dynamic time-varying Rayleigh fading channels without compromising the strict PLS requirements.

Using the same method, we analyze the computational complexity of conventional HRL methods [25]. Existing HRL approaches critically rely on the discretization of the continuous action space to formulate their lower-level decision-making. Assuming the transmit power is uniformly discretized into *M* discrete levels, the combinatorial action space for one active transmitter and |J| jammers scales exponentially as $O(M^{1+|J|})$. Consequently, the online execution complexity of conventional HRL severely expands to $O(D_{s}H+LH^{2}+H\cdot M^{1+|J|})$. This exponential growth introduces a dilemma: employing coarse discretization (a small *M*) inevitably leads to sub-optimal power allocation and massive performance degradation, whereas fine-grained discretization (a large *M*) triggers the curse of dimensionality, rendering real-time computation entirely infeasible. By deploying an Actor network to directly output continuous actions linearly scaled at O(1+|J|), our proposed CHRL elegantly sidesteps this discrete combinatorial explosion, guaranteeing both theoretically optimal power allocation and strict computational feasibility for real-time 6G deployments.

## 5. Numerical Results

**Figure 5:**
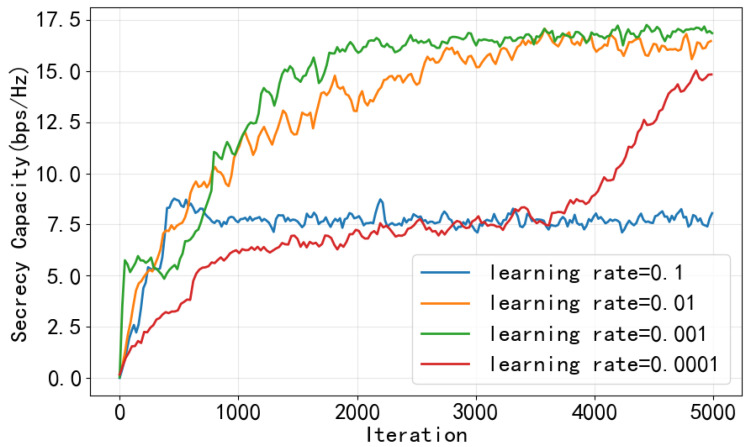
Sum secrecy capacity (SC) under different learning rates.

**Figure 6:**
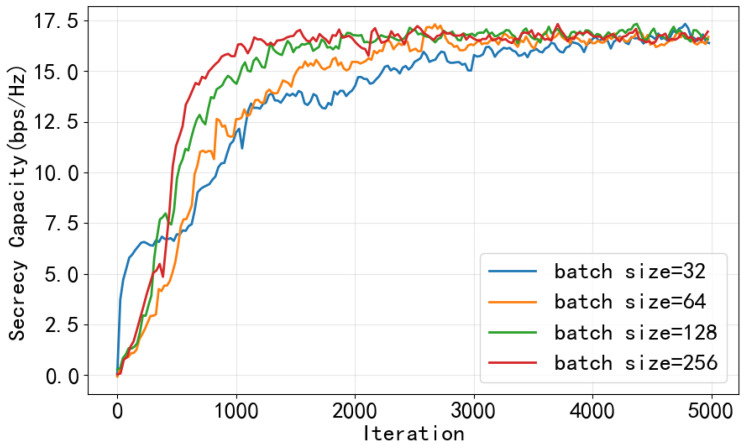
Sum SC under different batch sizes.

**Figure 7:**
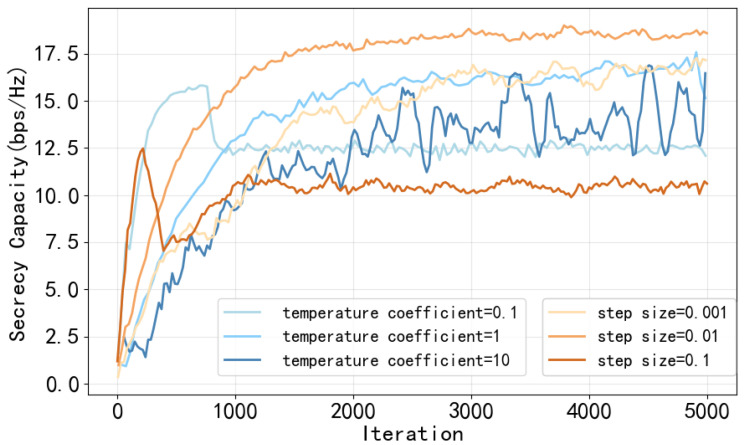
Sum SC versus the temperature coefficients and update step sizes of the Lagrange multiplier.

**Figure 8:**
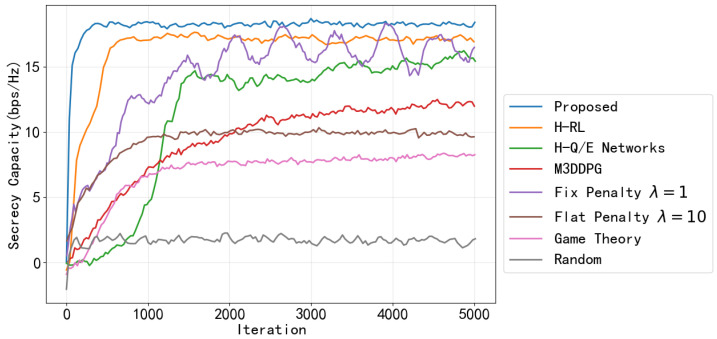
Sum SC using different methods.

In this section, we evaluate the performance of the proposed CHRL framework. The setting of simulation parameters is shown in the Table 1. The curves presented from Figure 5, Figure 6, Figure 7 and Figure 8 are based on actual training data obtained from a single training run, and a moving average with a window size of 50 iterations is applied to the raw training data.

First of all, the learning rate of the optimizer updating network parameters should be set to an appropriate value. As shown in Figure 5, if the learning rate is too large (e.g., 0.1), it may cause the model to converge to a local optimum. Conversely, if the learning rate is too small, the convergence process will be significantly slower. For instance, with a learning rate of 0.0001, the model only converges around the 5000th iteration. We ultimately set the learning rate to 0.001 for the subsequent simulations.

The influence of different batch sizes on the convergence performance during training is investigated in Figure 6. It can be observed that a small batch size fails to utilize all the data stored in the experience buffer and leads to slow convergence. In contrast, a large batch size, for instance, 256, results in the fastest convergence speed, albeit at the cost of increased training time per epoch.

To ensure the reliability of the proposed CHRL framework in practical deployments, it is imperative to investigate the robustness of key parameters, specifically the temperature coefficient τ in the upper-level Boltzmann policy and the update step size $\eta_{\lambda }$ for the lower-level Lagrange multipliers. Improper tuning of these parameters can disrupt the balance between reward maximization and risk avoidance, potentially trapping the network in sub-optimal local minima.

Figure 7 quantitatively demonstrates the sum SC versus iterations under various parameter settings.

The blue curves in Figure 7 demonstrate that the parameter τ orchestrates the trade-off between maximizing the expected reward and minimizing the constraint violation risk. An excessively large temperature (τ=10) causes the agent to over-explore, resulting in high variance and severe oscillations throughout the training process. Conversely, if τ is too small (τ=0.1), the policy becomes overly greedy. In this scenario, the agent is severely inhibited from exploring potentially high-reward node combinations if the E-network marginally overestimates their risk during early iterations, causing a premature convergence to a sub-optimal topology, approximately 12.5 bps/Hz. The fine-tuned value of τ=1 optimally balances initial thorough exploration with subsequent stable exploitation.

The orange curves in Figure 7 indicate that the step size $\eta_{\lambda }$ dictates the sensitivity of the continuous power allocation policy to security constraint violations. When $\eta_{\lambda }$ is excessively large ($\eta_{\lambda }=0.1$), a single security outage causes the penalty term to surge drastically. This massive penalty overrides the primary objective of maximizing SC, forcing the agent into a state of excessive inhibition. The agent learns to apply extreme, conservative continuous actions to avoid any conceivable risk, causing the sum SC to plummet sharply early in the training and lock into a sub-optimal baseline 10.5 bps/Hz. Alternatively, a very small step size ($\eta_{\lambda }=0.001$) causes the penalty to update too slowly, dragging down the convergence speed. The empirically chosen value of $\eta_{\lambda }=0.01$ provides the optimal robustness, allowing the agent to gracefully learn the safety boundaries while achieving the highest stable sum SC 18.5 bps/Hz.

We then compare the proposed method against seven baselines: (1) HRL [25], (2) H-Q/E networks [23], (3) Flat M3DDPG [29], (4) fixed penalty with a small weight λ=1, (5) fixed penalty with a large weight λ=10, (6) game theory [6] and (7) random. As shown in Figure 8, in the conventional fixed penalty baseline, a static penalty weight λ is directly incorporated into the reward function to penalize security constraint violations instead of using Lagrange multipliers. The reshaped reward $R_{t}$ is formulated as $R_{t}=r_{t}-\lambda \cdot \sum_{i=1}^{C}c_{i,t}$, where $r_{t}$ is the objective reward Equation (20) and $c_{i,t}$ is the immediate security cost Equations (21)–(23).

The simulation results in Figure 8 demonstrate that the proposed method consistently achieves the highest cumulative scores, stabilizing around 18.5 bps/Hz after approximately 1000 iterations. H-RL and H-Q/E networks follow closely, plateauing at 17.5 bps/Hz and 15.8 bps/Hz, respectively. The non-hierarchical M3DDPG struggles severely with the combinatorial explosion of the hybrid action space, converging extremely slowly to a sub-optimal 12.4 bps/Hz, which firmly validates the necessity of stratification. Furthermore, replacing our dynamic Lagrange method with static penalty terms proves highly ineffective. The fixed penalty with small λ baseline exhibits severe oscillations. It prioritizes short-term rewards but suffers from frequent security outages, failing to maintain a stable positive SC. Conversely, the fixed penalty with large λ baseline induces excessive inhibition, forcing the agent into overly conservative power allocation strategies that lock the performance at a severely degraded 10.0 bps/Hz. Other conventional methods, such as the game theory and random baseline, show gradual improvement but remain significantly lower. Overall, the demonstrated figure shows the superior convergence and secrecy performance of the proposed approach in this iterative task.

**Figure 9:**
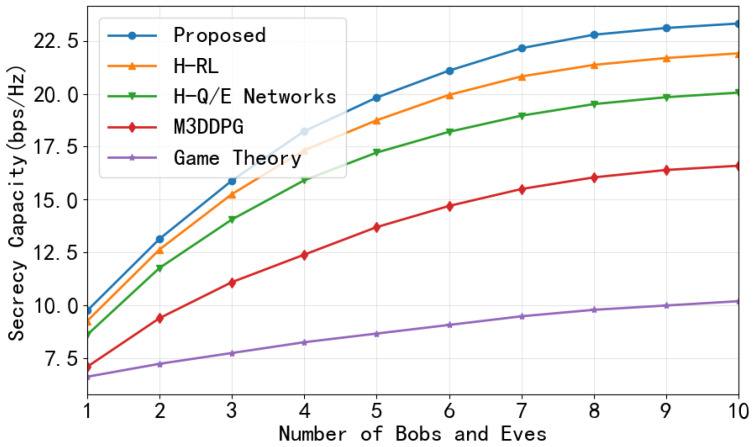
Sum SC versus number of Bobs and Eves.

Next, we investigate the impact of varying the number of Bobs and Eves. As shown in Figure 9, as the number of Bobs and Eves increases simultaneously from 1 to 10, the sum SC exhibits a monotonically increasing trend for all schemes, driven by the spatial multiplexing gains of serving more users. The proposed CHRL framework consistently outperforms the baselines, rising from approximately 10 to 23 bps/Hz and maintaining a significant performance gap over H-RL, H-Q/E networks, M3DDPG, and the game theory approach. Notably, the growth rate for the game learning-based methods gradually slows down as the network becomes denser.

**Figure 10:**
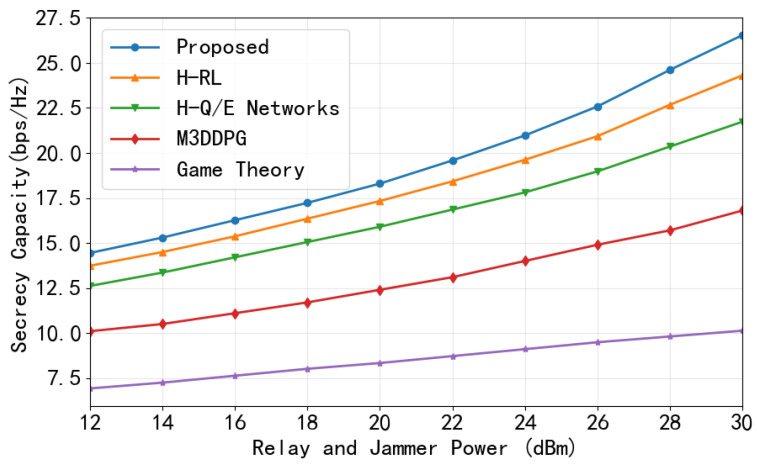
Sum SC versus relay and jammer transmit power.

To analyze the influence of node transmit power, we vary the power levels of relays serving Bobs and jamming nodes targeting. As shown in Figure 10, increasing the legitimate transmission power improves the legitimate channel capacity, thereby enhancing SC. The proposed CHRL framework consistently outperforms the baselines, exhibiting a steeper growth curve that reaches approximately 26.5 bps/Hz at 30 dBm, compared to roughly 24 bps/Hz for H-RL, 22 bps/Hz for H-Q/E networks, and 17 bps/Hz for M3DDPG.

**Figure 11:**
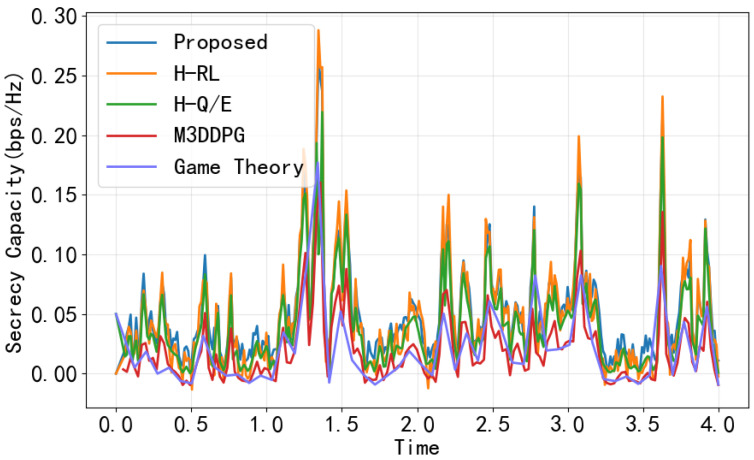
SC versus time under different methods.

To evaluate the efficacy of the proposed CHRL framework in satisfying security requirements, Figure 11 illustrates the instantaneous SC over time across various methods under identical channel realizations. All four methods exhibit similar trends, but only the proposed method and H-Q/E, with its added constraint, maintain strictly positive SC throughout the transmission. In contrast, M3DDPG and game theory methods intermittently fall below zero, failing to guarantee persistent security. Notably, the game-theoretic approach proves inadequate in this highly dynamic environment. The lack of an adaptive learning mechanism prevents it from performing rapid, real-time adjustments in response to the fast-fading channel coefficients modeled by the SDEs.

**Figure 12:**
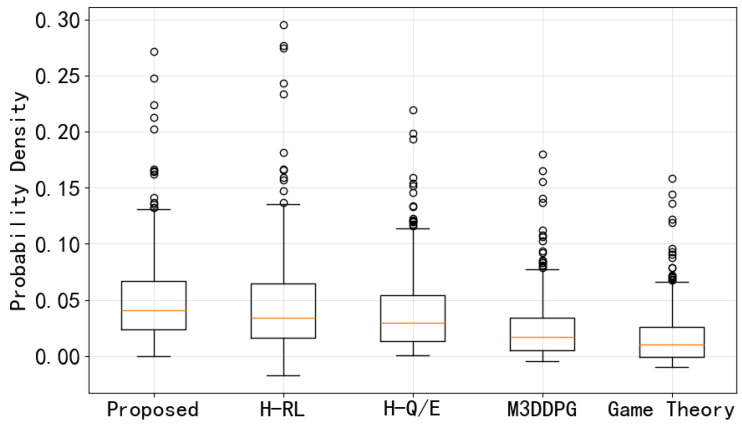
Boxplot comparison of SC distributions. The orange line within each box represents the median value, and the dots indicate outliers.

The boxplot based on Figure 11 is shown in Figure 12, which provides a statistical summary of the instantaneous SC distribution for each method. The proposed method exhibits the highest median value, demonstrating superior average performance compared to the baselines. Moreover, the distribution of the proposed method is strictly non-negative, with the lower whisker staying at or above zero, which validates the framework’s ability to strictly enforce the positive SC constraint. In contrast, H-RL, M3DDPG, and game theory methods show lower whiskers extending below zero, indicating frequent security outages where the SC becomes negative.

## 6. Conclusions

In this paper, we proposed a novel CHRL framework to address the challenge of secure cooperative communication in highly dynamic 6G networks. By modeling the time-varying wireless channels using SDEs, we established a realistic environment where traditional optimization methods often fail due to the lack of perfect CSI. This study offers several novel insights into PLS. First, we demonstrated that hierarchically decoupling the mixed-integer optimization problem fundamentally resolves the dilemma between the curse of dimensionality and performance degradation caused by action space discretization. Second, we shifted the traditional paradigm of merely maximizing SC to providing strict security guarantees. By utilizing a risk-aware meta-controller that models secrecy violations as long-term risks, the system ensures a strictly positive SC even under highly dynamic channel variations. Finally, we revealed the double-edged-sword nature of cooperative jamming. By framing continuous power allocation as a constrained zero-sum game and deploying a constrained M3DDPG algorithm against worst-case adversarial perturbations, our framework achieves unprecedented robustness. The simulation results confirm that the proposed method outperforms existing baselines, achieving SC improvements of approximately 6% over H-RL, 17% over H-Q/E networks, and 49% over M3DDPG, while the integration of Lagrangian multipliers rigorously prevents security outages.

## Figures and Tables

**Table 1:** Experimental parameter settings.

Parameter	Symbol	Value
Candidate intermediate nodes	*K*	100
Number of Bobs	*N*	4
Number of Eves	*Q*	4
SDE channel paths	-	100
SDE drift and initial parameters	$B,I_{0},Q_{0}$	1, 0, 0
Max transmit power (relays/jammers)	$P_{max,u},P_{max,j}$	20 dBm
Normalized noise variance	$\sigma^{2}$	1
Independent channel realizations	-	$10^{3}$
Communication duration	*T*	4s
Time scale of upper controller	*n*	10
Learning rate of optimizer	α	0.001
Batch size	*B*	256
Temperature coefficient of Boltzmann policy	τ	1
Update step size of Lagrange multipliers	$\eta_{\lambda }$	0.01
Hidden layers of neural network	*L*	3
Number of neurons in each layer	*H*	256

## Data Availability

Data is contained within the article.
